# Self-Reported Assessment of the Socio-Economic Impact of Anticancer Chemotherapy-Related Neurotoxicity

**DOI:** 10.3390/toxics11020104

**Published:** 2023-01-22

**Authors:** Guido Cavaletti, Alessia D’Acunti, Alessandro Porcu, Gabriella Masiello, Laura Del Campo, Gianfranca Traclò, Francesco De Lorenzo, Davide Paolo Bernasconi

**Affiliations:** 1 Experimental Neurology Unit, School of Medicine and Surgery, University of Milano-Bicocca, 20900 Monza, Italy; 2 Aimac—Italian Association of Cancer Patient, 00187 Roma, Italy; 3 Bicocca Bioinformatics Biostatistics and Bioimaging Center—B4, School of Medicine and Surgery, University of Milano-Bicocca, 20900 Monza, Italy; 4 Functional Department for Higher Education, Research, and Development, ASST Grande Ospedale Metropolitano Niguarda, 20162 Milan, Italy

**Keywords:** chemotherapy, neurotoxicity, self-reported, social impact, economic impact, health status

## Abstract

Chemotherapy-induced neurotoxicity is a well-known complication of several very effective systemic anticancer treatments, mainly presenting as cognitive impairment (“chemo-brain”) and peripheral neuropathy. The social and economic effects of long-lasting chemotherapy-induced neurotoxicity on patients’ lifestyles and their relationships are under-investigated, and their impact is, therefore, largely unknown. In this study, we used a web-based questionnaire to record the self-reported perception of chemotherapy-induced neurotoxicity on cancer patients’ health status, but also on several different aspects of their daily life. From the study results, it emerged that the impact of chemotherapy-induced neurotoxicity on personal, social, and working activities is very high. A similar effect was also observed when the psychological impact is assessed. Moreover, there is evidence suggesting that the management of CIPN is suboptimal; this is partially due to a lack of effective drugs, but also of appropriate advice from healthcare providers. In conclusion, this study provides evidence for the relevance of the impact on the explored aspects of the daily life of cancer patients and spotlights the need for a larger and more structured investigation on these long-term side effects of anticancer chemotherapy.

## 1. Introduction

Chemotherapy-induced neurotoxicity (CIN) is a well-known complication of several very effective systemic anticancer treatments, mainly presenting as cognitive impairment (“chemo-brain”) [1] and peripheral neuropathy (CIPN) [2], and no effective treatment is available for either of these conditions [3,4]. In clinical practice, CIN occurrence and its manifestations tend to be closely monitored during chemotherapy (also because they can represent dose-limiting side effects), but their presence is generally less carefully assessed in the follow-up, despite the fact that they can potentially have a relevant impact on cancer survivors’ long-term quality of life [5,6,7,8,9,10]. Moreover, even less well established are the social and economic effects of CIN on patients’ lifestyles and their relationships. In fact, within this context, it is very important to recognize the importance of tertiary prevention and ensure it to people with a previous cancer diagnosis. More precise knowledge of these long-term aspects is crucial, particularly given the increasing number of cancer patients receiving effective treatments allowing them to be cured and to achieve a normal life expectancy or, at least, to gain a very long survival and active life.

Patient-reported outcome measures (PROMs) are increasingly utilized to assess the severity of CIN, particularly to evaluate CIPN [11,12], since they represent the most suitable and reliable method to capture the individual perception of the severity of CIN-induced events on health status. However, this patient-centered approach has not yet been extended to other domains of patients’ daily life.

In this study, we used a web-based questionnaire posted on the website of a representative oncology patients’ community to record the self-reported perception of CIN of cancer patients on their health status, but also on several different aspects of their daily life.

## 2. Materials and Methods

The questionnaire used in this study was created by a panel composed of physicians, experts in CIN, and “Associazione italiana malati di cancro” (Italian Association of Cancer Patients—AIMaC) members, including cancer patients.

After this initial activity, the questionnaire and the study plan were submitted to the University of Milano-Bicocca Ethics Committee for approval (granted with n. 650, 22 January 2022).

The questionnaire included questions on patients’ general demographic data, cancer-related medical history, and personal assessment of CIN’s impact on their health status, activities of daily life, working activities, and neurotoxicity management (see Supplementary Material File S1 for the full version of the questionnaire). The questionnaire was advertised through the Aimac periodic newsletter (more than 3000 subscribers) and posted on the association website (https://www.aimac.it/), soliciting cancer patients to anonymously complete it as had already done in previous campaigns. In order to allow the largest number of cancer patients to provide their answers, they were considered eligible if aged 18 or older, if they received any neurotoxic anticancer chemotherapy, and were able to autonomously complete the online web-based survey. The questionnaire was left online for 3 months (22 March 2022–22 June 2022) and it was introduced by a text where it was clearly explained, in lay-people language agreed with Aimac, that all the questions were exclusively related to central and/or peripheral neurotoxicity ensuing after chemotherapy, and also describing their main symptoms.

The study fulfilled the STROBE guidelines for cross-sectional studies (available at: https://www.strobe-statement.org/ accessed on 5 January 2023), except for the following items:

- Item #8 was not fully applied, since the questionnaire was not validated
- Item #10: study size was not predefined, since the questionnaire was freely available and the number of responders was not predictable
- Item #12 was only partly applied, given the type of study
- Items #13 and #17 were not applicable

## 3. Results

The first and the second sections of the questionnaire described in lay-people language the main symptoms of CIPN and CIN, and specific questions were asked, thus allowing the responder to be guided and the assessor to properly check if she/he really suffered from CIPN and/or CIN.

The page with the questionnaire notice was viewed 2797 times, and the page with the questionnaire was viewed 500 times. A total of 162 patients completed the questionnaire (12.3% males, and 87.7% females, reflecting the predominance of breast cancer among the responders).

Most of the patients were aged 51–60 (38.9%), and the majority of them had a medium-to-high level of education. Regarding their cancer history, approximately half of the responders (56.8%) were in remission, with a long history of cancer (54.9% of the responders in the range 2–5 years), and 61.7% of them were off treatment. More than half of the responders had breast cancer (see Table 1).

### 3.1. Results Divided according to Their Domains

#### 3.1.1. Health Status

**Figure 1:**
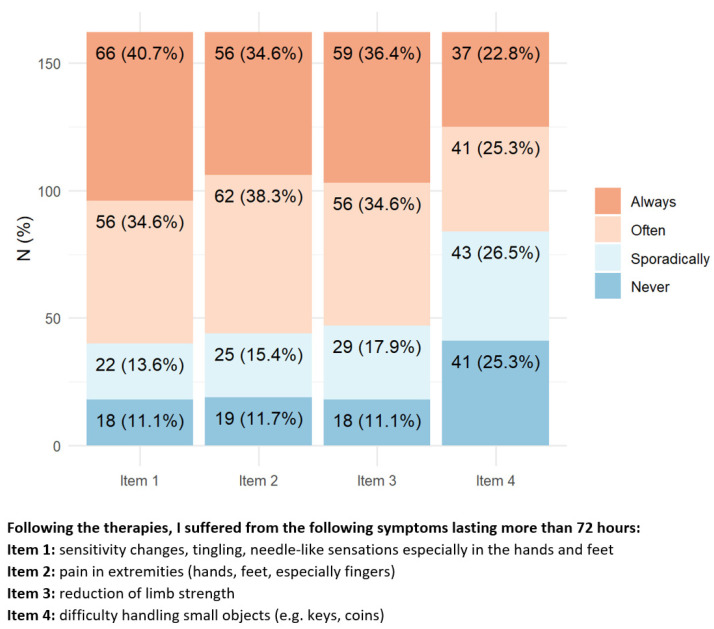
Items with the highest percentage of positive answers indicating a relevant impact of peripheral neurotoxicity on health status.

Regarding the presence of CIPN symptoms (Figure 1), approx. 70% of responders reported having always/often alterations in sensation, distal pain, and reduced arm strength. Nearly 50% of them reported having always/often difficulty in common daily tasks such as manipulating small objects, climbing one flight of stairs, and approximately a quarter of them had problems in walking in dark rooms.

By contrast, only a minority of responders reported difficulty in swallowing, speaking, or having visual impairment.

**Figure 2:**
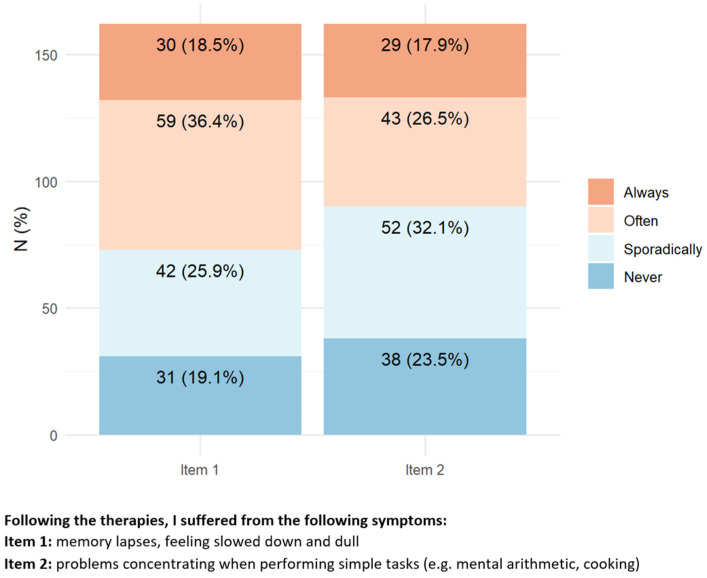
Items with the highest percentage of positive answers indicating a relevant impact of central neurotoxicity on health status.

Regarding central nervous system neurotoxicity symptoms (Figure 2), approximately 40–50% of patients reported experiencing always/often difficulty in memory or concentration while performing simple tasks (e.g., cooking, mental calculation).

#### 3.1.2. Personal and Social Impact

**Figure 3:**
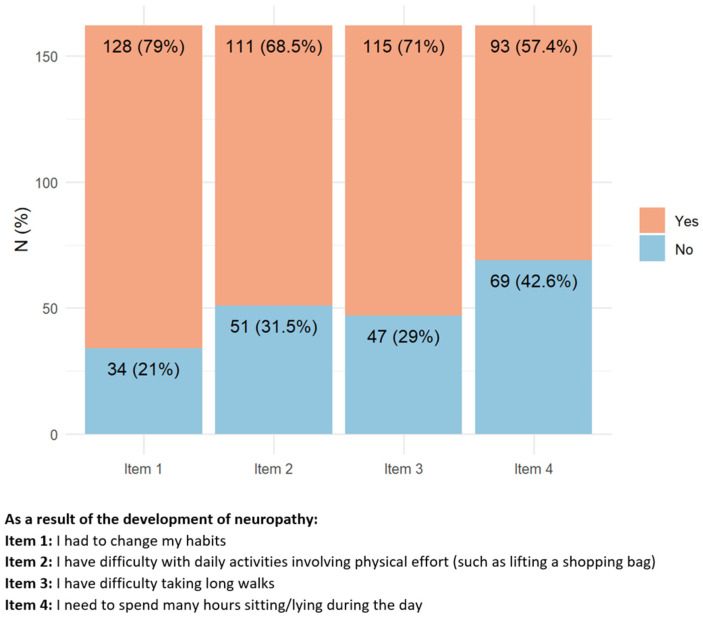
Items with the highest percentage of positive answers indicating a relevant social and personal impact of peripheral neurotoxicity.

According to the questionnaire answers, only 4.3% of the responders needed assistance with eating, dressing, and personal hygiene. However, the majority of responders had to change their habits (e.g., they reported difficulties in performing common activities requiring physical fitness such as carrying a heavy shopping bag and being unable to walk for a relatively long distance easily achievable before chemotherapy), 57.4% reported feeling the need to sit or rest in bed several hours during the day, and 71% reported sexual problems ensuing after chemotherapy, without any relevant association with the tumor site (Figure 3). Moreover, 26.5% of responders reported being unable even to walk for a short distance (e.g., taking the dog for a walk, or going to buy the newspaper).

#### 3.1.3. Working Impact

**Figure 4:**
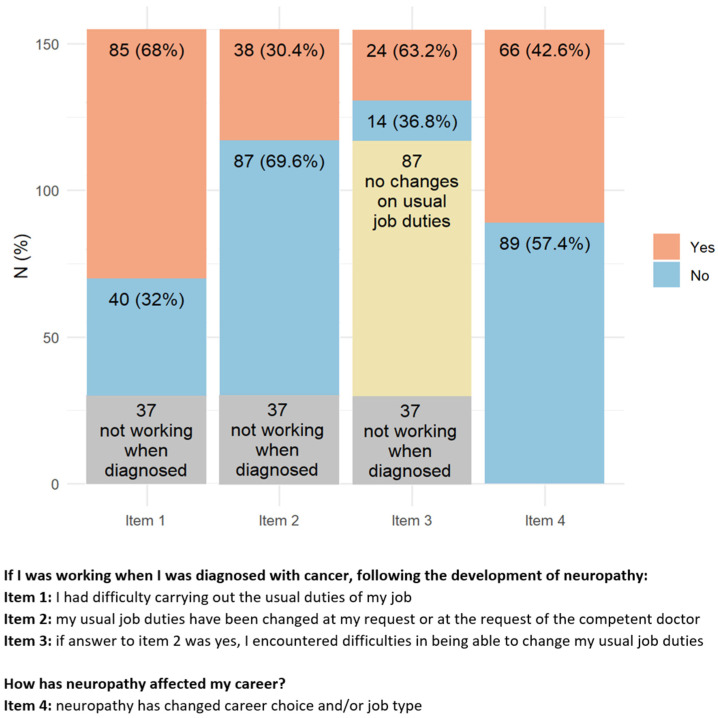
Items with the highest percentage of positive answers indicating a relevant working impact of peripheral neurotoxicity. Regarding Item 3, in yellow are active workers who did not changed their usual job duties, in blue those who changed without difficulties, and in red those with difficulties.

Most of the responders (77.2%) were active workers at the moment of cancer diagnosis (Figure 4). After cancer treatment, 68% of them reported having difficulties due to CIPN symptoms in performing their usual working duties, 30.4% had to change their working activity, and for 63.2% of them, this change was troublesome. Despite this not being the case for the majority of responders, 42.6% of them modified their working plans, 28.4% felt that CIPN presence hampered the development of their career, 35.5% had to move from full to part-time, and 25.6% reported hostile behaviors due to their health conditions. A minority of responders (12.9%) attributed work loss to the presence of CIN.

#### 3.1.4. Psychological Impact

**Figure 5:**
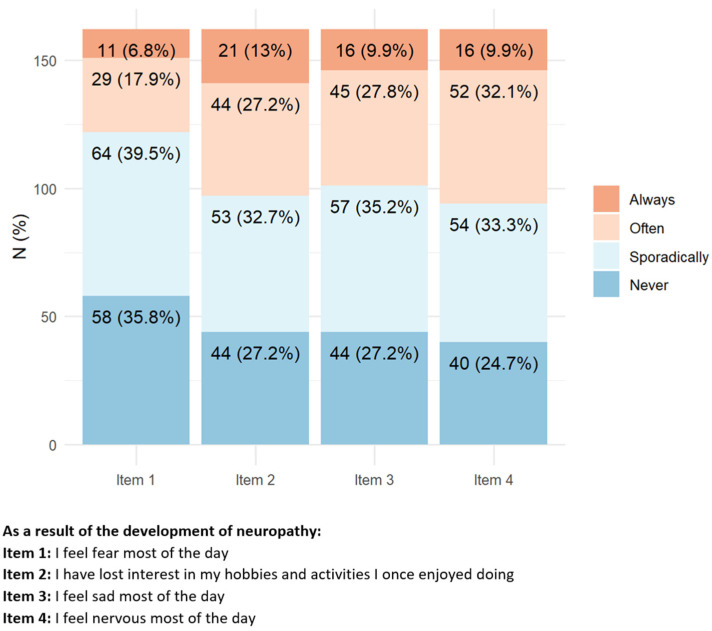
Items with the highest percentage of positive answers indicating a relevant psychological impact of peripheral neurotoxicity.

More than 80% of the responders reported having a very positive attitude toward their ability to deal with cancer (Figure 5). However, several responders reported suffering always/often from mood changes (mainly irritability, sadness, fear, and loss of interest in hobbies). A smaller proportion of the responders (20–30%) felt always/often to be socially distant from their family/relatives, to feel loneliness, and, overall, were “angry” for most of the day.

#### 3.1.5. Neurotoxicity Management

Once asked regarding their opinion on the management of their CIPN, only 42.6% of the responders reported that they received useful and detailed information; among them, 63.9% were informed by the hospital medical team, while only 9.3% of the responders were assisted by their general practitioner, despite most of them being no longer under hospital-based follow-up. Regarding CIPN treatment, 44.4% of the responders received some drug, and only approximately 20% were prescribed physical rehabilitation or alternative therapies (in most cases acupuncture, gymnastic yoga, or non-prescription substances).

## 4. Discussion

This exploratory study was designed to detect largely under-assessed features of long-term (i.e., persisting after treatment withdrawal) chemotherapy neurotoxic side effects [5,6,13,14], and the importance of its results are closely linked to the impact of these side effects on several relevant aspects of cancer patients’ daily life.

The responders feedback obtained by the questionnaire was in line with previous campaigns organized by Aimac (e.g., a questionnaire investigating the impact of the SARS-CoV-2 pandemic on cancer patients left online in the period April 2020–April 2021 received 540 answers), thus confirming the interest of the association affiliates on the topic of neurotoxicity.

The remarkable impact of CIN on the individual health of cancer survivors is confirmed by the answers collected in the study, and these data are in line with previous studies [15,16]. Remarkably, most of the responders had a breast cancer diagnosis and a tumor treated with drugs (namely taxanes) reported a high incidence of CIN [15].

However, what emerges very clearly from the study is that the impact of CIN on personal, social, and working activities is also very high. A similar effect was also observed when the psychological impact is assessed. Moreover, there is evidence suggesting that the management of CIPN perceived by the responders is suboptimal, a result partially due to a lack of effective drugs, but also lack of appropriate advice from healthcare providers.

The study has some weaknesses, mainly represented by the relatively low number of patients who completed the questionnaire, the unbalanced mix of cancers, the absence of specific assessments to rule out possible confounding factors (e.g., depression), and a possible selection bias inducing mainly those patients with the most relevant impact of CIN on their lifestyle to complete the questionnaire. The need to better understand the link between specific treatments and side effects, based on different chemotherapies specific per cancer organs, as well as re-habilitation costs for the National Health System, also remains. Moreover, definite results on the socio-economic impact of anticancer chemotherapy neurotoxicity should be achieved through more formal, case–control trials, and a multi-national approach would also be advisable, since, for instance, different levels of social support in different countries might produce variable levels of impact on cancer patients. However, the study’s major strengths are the involvement of patients in questionnaire development and the novelty of the results, since this is the first study specifically investigating the socio-economic impact of anticancer chemotherapy neurotoxicity using a self-reported assessment method.

## 5. Conclusions

Our study should not be considered as the final answer to the unsettled issue of the socio-economic impact of anticancer chemotherapy-related neurotoxicity, but it is rather an exploratory study, strongly highlighting the need for a more formal and structured analysis. In fact, only the healthcare costs and work loss burden of patients with CIPN has been formally investigated, showing that average healthcare costs were USD 17,344 higher for CIPN cases than their non-CIPN controls, with outpatient costs being the highest component [17].

Our study based on the self-assessment of the impact of CIN on cancer survivors’ lifestyles provides initial evidence of the relevance of the issue and spotlights the need for larger and more structured investigation of these long-term side effects of anticancer chemotherapy to gain more precise knowledge of the real burden of limitations associated with the use of neurotoxic drugs.

Moreover, the study perfectly fits into the 7th Recommendation of the Report of the Mission Board for Cancer “Conquering Cancer: Mission Possible”, which aims at developing an EU-wide research program and policy support to improve the quality of life of cancer patients and survivors, family members and carers.

## Figures and Tables

**Table 1:** Clinical and demographic data of the responders.

		N (%)
Gender	F	142 (87.7)
	M	20 (12.3)
Age, y	0–20	1 (0.6)
	21–40	6 (3.7)
	41–50	42 (25.9)
	51–60	63 (38.9)
	61–70	41 (25.3)
	71+	9 (5.6)
Education *	Primary	1 (0.6)
	Lower secondary	10 (6.2)
	Upper secondary	90 (55.6)
	Bachelor or superior	59 (36.4)
	No answer	2 (1.2)
Years from cancer diagnosis	0–1	42 (25.9)
	2–5	89 (54.9)
	6–9	18 (11.1)
	10+	13 (8.0)
Current status	Long-term survivor/follow-up	94 (58.0)
	Under treatment	67 (42.0)
Cancer site	Breast	89 (54.9)
	Gastro-intestinal	19 (11.7)
	Hematological	18 (11.1)
	Gynecological	12 (7.4)
	Bladder	10 (6.2)
	Other sites	14 (8.6)

* according to International Standard Classification of Education (ISCED, available at: https://ec.europa.eu/eurostat).

## Data Availability

Data supporting reported results can be found at (https://board.unimib.it/datasets/jhmg2n63g2 accessed on 9 January 2023 or https://doi.org/10.17632/jhmg2n63g2.2).

## Supplementary Materials

The following supporting information can be downloaded at: https://www.mdpi.com/article/10.3390/toxics11020104/s1, File S1: Survey on The Impact of Neuropathies on Patients’ Lives.
