# Supplementary material for: Self-Reported Assessment of the Socio-Economic Impact of Anticancer Chemotherapy-Related Neurotoxicity
Source: Toxics. 2023 Jan 22;11(2):104. doi: 10.3390/toxics11020104 (PMC9966709; doi:10.3390/toxics11020104)
Supplement: Supplementary file 1 [file toxics-11-00104-s001.zip › toxics-2106523-supplementary.pdf]

SURVEY ON THE IMPACT OF NEUROPATHIES ON PATIENTS' LIVES

Cancer patients are prone to neurological complications that can have a negative effect on quality of life. Some are directly attributable to the disease, others, the most frequent, are the result of the side effects of some therapies.

The aim of this survey is to investigate the impact that the development of a neuropathy had on your life.

The questionnaire is completely anonymous, and it takes about 10 minutes to complete.
For any doubt or question, you can contact Aimac on the telephone number 06 4825107, from Monday to Friday from 09:00 am to 7:00 pm, or send an email using the appropriate form on the website www.aimac.it/scrivici.

There is a version of this questionnaire specifically dedicated to caregivers, i.e. people, family or friends, who take care of the patient. At the end of the questionnaire you will be given a link and a code. If you want, you can invite the person who assists you in your treatment journey to participate in the survey by providing them with that link and that code.

FIRST PART
Patient information

Gender: (Only one answer is possible)

1. |__| Male	2. |__| Female	99. |__| I do not answer or other

Age: (Only one answer is possible)

1. |__| 0-20 years	2. |__| 21-40 years	3. |__| 41-50 years
4. |__| 51-60 years	5. |__| 61-70 years	6. |__| 71 years or older

Nationality: (Only one answer is possible)

1.|__| Italian	2.|__| Country within European Union	3.|__| European country extra-EU
4.|__| Asian	5.|__| African	6.|__| Latin America
7.|__| North America	8. |__| Australian	99.|__| I do not answer

Province of residence in Italy:|__|__|

Education degree (Only one answer is possible)

1.|__|None	2.|__|Primary school	3.|__|Secondary school
4.|__|High school	5.|__| Bachelor degree or higher	99.|__| I do not answer

Current tumor status (Only one answer is possible)

1.|__|Primary tumor	2.|__|Recurrence	3.|__|With metastases	4 .|__|Remission
99.|__|I do not know / I do not answer			

How long has it been since diagnosis? (Only one answer is possible)

1. |__| 0-1 year 2. |__| 2-5 years 3. |__| 6-9 years
4. |__| 10 years or more

At this moment, my current status in the treatment path is: (Only one answer is possible)

1. |__| Under treatment (surgery, chemo/radiotherapy, ormon therapy, pain control therapy, etc)
2. |__| Involved in a clinical trial
3. |__| Under palliative care
4. |__| Follow-up
5. |__| Long term survivor (free from disease and treatment since at least 5 years)
99.|__| I do not know / I do not answer

At this moment, I am: (Only one answer is possible)

1. |__| Hospitalized
2. |__| On a day hospital/outpatient basis
3. |__| Under home care
4. |__| At home (*without treatments, only follow-up visits*)
5. |__| In hospice
99.|__| I do not know / I do not answer

Primary tumor site: (Only one answer is possible)

1. |__| Oral cavity 2. |__| Colon 3. |__| Esophagus 4. |__| Pharynx 5. |__| Liver
6. |__| Breast 7. |__| Nose 8. |__| Ear 9. |__| Bone 10. |__| Ovary
11. |__| Pancreas 12. |__| Skin 13. |__| Penis 14. |__| Pleura 15. |__| Lung
16. |__| Prostate 17. |__| Kidney 18. |__| Rectum 19. |__| Hematopoietic system (e.g. Lymphoma, Leukemia, Myeloma, Etc...)
20. |__| Central Nervous System 21. |__|Neuroendocrine System 22. |__| Stomach
23. |__| Soft Tissue 24. |__| Testicle 25. |__| Thymus
26. |__| Thyroid 27. |__| Uterus 28. |__| Vagina
29. |__| Bladder 30. |__| Bile ducts 31. |__| Vulva
32. |__| Other (specify_____________________________________)
99. |__| I do not know / I do not answer

Medications I am currently taking: (Multiple answers are possible)

1. |__| Cisplatin 2. |__| Carboplatin 3. |__| Oxaliplatin 4. |__| Paclitaxel
5. |__| Docetaxel 6. |__| Vincristine 7. |__| Vinblastine 8. |__| Bortezomib
9. |__| Talidomide 10. |__| Other (specify_____________)
99. |__| I do not know / I do not answer

SECOND PART

Effects of treatment on the nervous system

This part is divided into two sections intended to evaluate the effects of treatmenton your nervous system

Section 1

ALL THE FOLLOWING QUESTIONS REFER EXCLUSIVELY TO POSSIBLE EFFECTS THAT CANCER TREATMENT MAY HAVE ON YOUR PERIPHERAL NERVOUS SYSTEM ("NEUROPATHY")

Following the therapies, I suffered from the following symptoms lasting more than 72 hours:

Please, choose a value from 1 to 4, where 1 means Never, 2 Sporadically, 3 Often and 4 Always

- sensitivity changes, tingling, needle-like sensations especially in the hands and feet
  1-------2-------3-------4
- pain in extremities (hands, feet, especially fingers)
  1-------2-------3-------4
- reduction of limb strength
  1-------2-------3-------4
- difficulty swallowing solid food
  1-------2-------3-------4
- difficulty swallowing liquid food
  1-------2-------3-------4
- difficulty articulating speech
  1-------2-------3-------4
- vision reduction
  1-------2-------3-------4
- difficulty handling small objects (e.g. keys, coins)
  1-------2-------3-------4
- difficulty climbing a flight of stairs due to lack of strength
  1-------2-------3-------4
- difficulty climbing a flight of stairs due to unsteadiness
  1-------2-------3-------4
- difficulty walking in the dark
  1-------2-------3-------4

Section 2

THE FOLLOWING QUESTIONS REFER TO OTHER POSSIBLE EFFECTS THAT THE CANCER TREATMENT MAY HAVE INDUCED ON YOUR CENTRAL NERVOUS SYSTEM

Following the therapies, I suffered from the following symptoms:

Please, choose a value from 1 to 4, where 1 means Never, 2 Sporadically, 3 Often and 4 Always

- memory lapses, feeling slowed down and dull
  1-------2-------3-------4
- problems concentrating when performing simple tasks (e.g. mental arithmetic, cooking)
  1-------2-------3-------4

Please, answer Yes or No

- inflammation of the spinal cord (transient myelopathy)
  1.|__|Yes 2.|__|No
- necrosis of a part of the brain
  1.|__| Yes 2.|__|No
- other (If Yes, please specify)
  1.|__| Yes (specify ____________________) 2.|__|No

THIRD PART
Psycho-social aspects
This part is divided into three sections intended to evaluate the consequences of the adverse effects of the treatment on different aspects of your life

<u>Section 1</u>
THE FOLLOWING QUESTIONS REFER TO ASPECTS CONCERNING CONSEQUENCES ON THE QUALITY OF YOUR LIFE

As a result of the development of neuropathy:

Please, answer Yes or No

- I had to change my habits
  1.|__| Yes 2.|__|No
- I have difficulty with daily activities involving physical effort (such as lifting a shopping bag)
  1.|__| Yes 2.|__|No
- I have difficulty taking long walks
  1.|__| Yes 2.|__|No
- I have difficulty even taking short walks (e.g. walking the dog, buying a newspaper close to home)
  1.|__| Yes 2.|__|No
- I need to spend many hours sitting/lying during the day
  1.|__| Yes 2.|__|No
- I need help with my daily activities (eating, dressing, washing or going to the bathroom)
  1.|__| Yes 2.|__|No
- I have relationship difficulties with my loved ones (partner/family/friends)
  1.|__| Yes 2.|__|No
- During sexual intercourse, I have difficulties that were not present before the therapies and/or the operation
  1.|__| Yes 2.|__|No

Section 2

THE FOLLOWING QUESTIONS REFER TO ASPECTS CONCERNING CONSEQUENCES ON YOUR JOB

Please, answer Yes or No

I was employed when I was diagnosed with cancer

1.|__|Yes 2.|__|No

As a result of the development of neuropathy:

Please, answer Yes or No

- I had difficulty carrying out the usual duties of my job
  1.|__|Yes 2.|__|No
- my usual job duties have been changed at my request or at the request of the competent doctor
  1.|__| Yes 2.|__|No
- if answer to previous point was yes, I encountered difficulties in being able to change my usual job duties
  1.|__| Yes 2.|__|No
- I had to deal with hostile attitudes due to my health conditions in my working environment
  1.|__| Yes 2.|__|No

How has neuropathy affected my career?

- neuropathy resulted in a lack of career advancement and/or promotion
  1.|__| Yes 2.|__|No
- neuropathy has changed career choice and/or job type
  1.|__| Yes 2.|__|No
- I work part-time instead of full-time
  1.|__| Yes 2.|__|No
- I lost my job
  1.|__| Yes 2.|__|No

Section 3

THE FOLLOWING QUESTIONS REFER TO ASPECTS CONCERNING CONSEQUENCES ON YOUR EMOTIONAL SPHERE

As a result of the development of neuropathy:

Please, choose a value from 1 to 4, where 1 means Never, 2 Sporadically, 3 Often and 4 Always

- I feel fear most of the day
  1-------2-------3-------4
- I have lost interest in my hobbies and activities I once enjoyed doing
  1-------2-------3-------4
- I regained interest in activities I loved but had neglected
  1-------2-------3-------4
- I feel sad most of the day
  1-------2-------3-------4
- I feel nervous most of the day
  1-------2-------3-------4
- I am grateful that I was able to deal with the disease
  1-------2-------3-------4
- I feel distant from my family and/or loved ones and friends
  1-------2-------3-------4
- I feel alone most of the day
  1-------2-------3-------4
- I feel angry most of the day
  1-------2-------3-------4

FOURTH PART

Neurotoxicity management

This part is intended to assess how you felt supported by healthcare professionals in dealing with adverse effects of therapy

As a result of the development of neuropathy:

Please, answer Yes or No

- I been provided with useful information to deal with it (e.g.: who to contact; what to do; physiotherapy, speech therapy, psychology, etc. services to contact, ...)
  1.|__|Yes 2.|__|No

  If yes, who provided the information? *(multiple answers are possible)*

  1. The hospital team who took care of me
  2. The general practitioner
  3. A private specialist
  4. A relative or friend
  5. Other, please specify: ____________________

- I was prescribed a drug treatment
  1.|__| Yes 2.|__|No
- I was prescribed a rehabilitation activity
  1.|__| Yes 2.|__|No
- I was recommended integrative therapies
  1.|__| Yes If yes, specify (e.g. acupuncture, yoga, … _____________) 2.|__|No

Thank you for taking the time to participate in this survey. There is a version of this questionnaire specifically dedicated to caregivers, i.e. people, family or friends, who take care of the patient. If you wish, you can invite the person who assists you in your treatment path to participate in the survey at this link: https://www.aimac.it/indagine-neuropatie-caregiver

In order to complete the questionnaire, your caregiver must enter the following code: XXX
